# P-1606. Utilizing Diagnostic Stewardship to Help Increase the Yield and Clinical Value of Broad Range PCR Testing at a Tertiary Children’s Hospital

**DOI:** 10.1093/ofid/ofae631.1773

**Published:** 2025-01-29

**Authors:** Sarah Fortna, Muayad Alali, Kirsten Prabhudas-Strycker, Kagan A Mellencamp, LaKeisha Boyd, Michael Goings, Haseeba Khan, Mackenzie Fahey, Matthew Samaro, Jack G Schneider

**Affiliations:** Indiana University School of Medicine, Indianapolis, Indiana; Indiana university, Carmel, Indiana; Indiana University, Indianapolis, Indiana; Indiana University, Indianapolis, Indiana; Indiana University, Indianapolis, Indiana; Indiana University, Indianapolis, Indiana; Indiana University School of Medicine, Indianapolis, Indiana; Indiana University School of Medicine, Indianapolis, Indiana; Indiana University, Indianapolis, Indiana; Indiana University School of Medicine, Indianapolis, Indiana

## Abstract

**Background:**

Broad range PCR testing (BR-PCR) in various clinical samples has the ability to provide timely diagnoses that cannot always be made through conventional testing (CT), yet its diagnostic yield and clinical impact have been variable, especially by specimen type. As such, we developed an ID-physician led diagnostic stewardship approval protocol to help optimize test usage.

**Methods:**

We conducted a single-center, retrospective pre/post study to assess the impact of an ID-led diagnostic stewardship approval protocol for BR-PCR testing. All clinical specimen types obtained for BR-PCR at Riley Hospital for Children were evaluated between 10/1/2019 to 4/30/2022 (pre-intervention) and 5/1/2022-12/31/2023 (post-intervention). Clinical relevancy of BR-PCR results was determined after review from two ID physician experts and compared between the two time periods, along with clinical impact and overall cost savings.

**Results:**

A total of 931 BR-PCR tests were sent from 238 specimens collected from 175 patients in the pre-intervention period, while 208 BR-PCR tests were sent from 65 specimens collected from 65 patients in the post-intervention period. Clinical relevancy of results was determined to be 30.7% and 56.9% for pre-and post-intervention periods, respectively (p< 0.001). 23.1% of post intervention results led to a change in clinical management, compared to 12.6% in the pre-intervention period (p=0.035). Bronchial lavage (BAL) was the most common specimen type with 52.9% of results being clinically relevant post-intervention, compared to 29.6% in the pre-intervention period; p=0.068). Results that led to a clinical change in management were also slightly higher but non-significant for BALs post-intervention (11.8% vs 9.9% pre intervention; p=0.816). Overall cost savings post-intervention was estimated to be $200,000.

**Conclusion:**

Use of an ID-physician led diagnostic stewardship approval protocol led to an overall improvement in clinical utility for BR-PCR testing at our institution and was influenced by specimen type. Prospective, multi-center studies are needed to determine which specimen types, diagnoses, and potential diagnostic stewardship measures will help increase the yield and clinical value of BR-PCR testing.

**Disclosures:**

**Jack G. Schneider, MD**, MiraVista Diagnostics: Advisor/Consultant

